# Supplementary material for: Structural insights into the mechanism of the membrane integral N-acyltransferase step in bacterial lipoprotein synthesis
Source: Nat Commun. 2017 Jul 4;8:15952. doi: 10.1038/ncomms15952 (PMC5500888; doi:10.1038/ncomms15952)
Supplement: Supplementary Information [file ncomms15952-s1.pdf]

Type of file: PDF
Size of file: 0 KB
Title of file for HTML: Supplementary Information
Description: Supplementary figures, supplementary tables and supplementary references.

Type of file: MOV
Size of file: 0 KB
Title of file for HTML: Supplementary Movie 1
Description: MDS of the LntEco active site. Exemplar molecular motions of residues Glu267, Lys335, Glu343 and Cys387 within the active site of LntEco. For much of the simulation the hydrogen of the cysteine side chain is involved in a hydrogen bond (dashed line) with the catalytic Glu267, suggesting that this residue is responsible for the formation of the thiolate intermediate. The neighbouring Lys335 and Glu343 form a salt bridge for the majority of the molecular simulation.

Type of file: MOV
Size of file: 0 KB
Title of file for HTML: Supplementary Movie 2
Description: MDS of LntEco with bound substrates and products. Example molecular dynamics of LntEco at different stages in the reaction: 1) Cys387 in thiol form with bound POPE, 2) Cys387 in thiolate form with bound POPE, 3) tetrahedral intermediate, 4) palmitoylated intermediate with bound lyso-PE, 5) palmitoylated intermediate, 6) palmitoylated intermediate with bound diacyl FSL-1, 7) tetrahedral intermediate, 8) Cys387 in thiol form bound triacyl FSL-1. In all cases, the protein is shown in a cartoon representation. Cys387, lipid and lipopeptide are shown in spheres. Lipid and peptide carbon atoms colored purple and grey, respectively.

Type of file: PDF
Size of file: 0 KB
Title of file for HTML: Peer review file
Description:

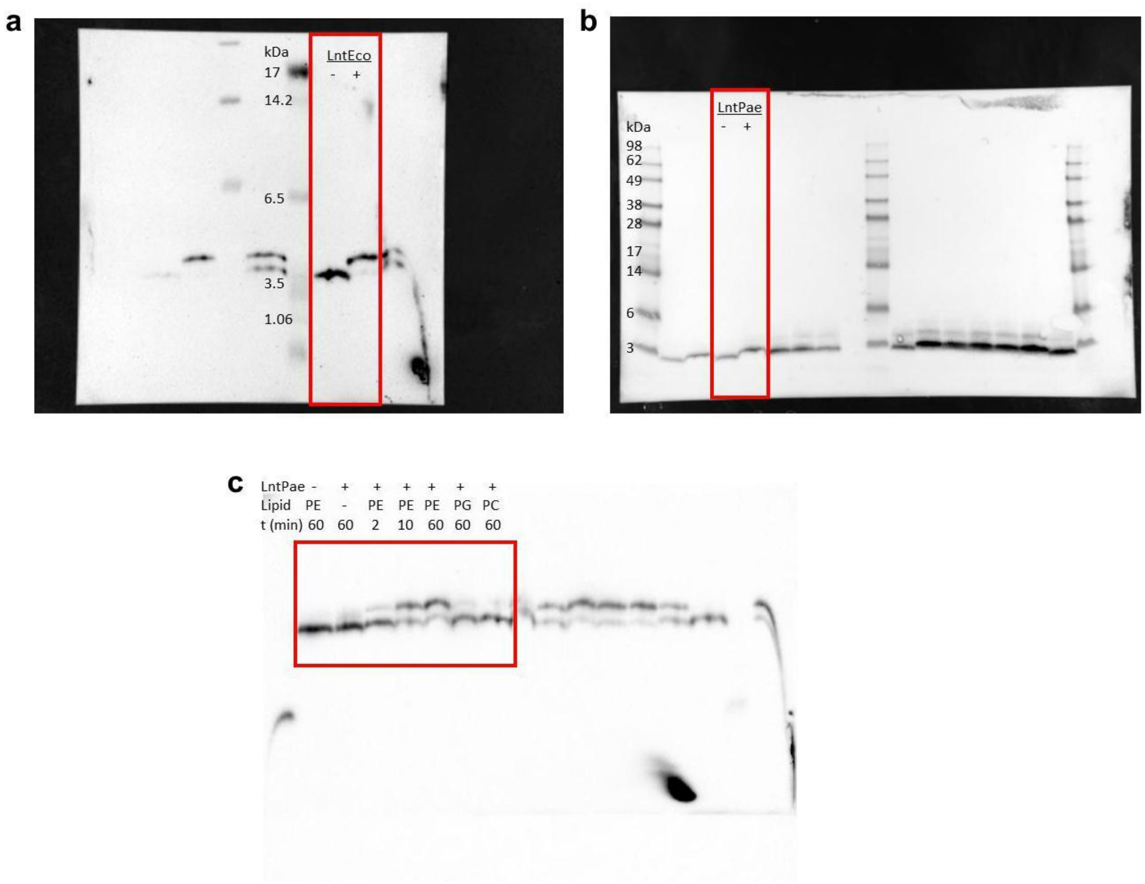

Supplementary Figure 1 | Photographic images of original western blots. (**a**) Uncropped image of western blot used for LntEco N-acyltransferase activity quantitation (**Fig. 2a**). (**b**) Uncropped image of western blot used for LntPae N-acyltransferase activity quantitation (**Fig 2a**). (**c**) Uncropped image of western blot in **Fig 2b.** Red boxes identify cropped sections in **Fig. 2**.

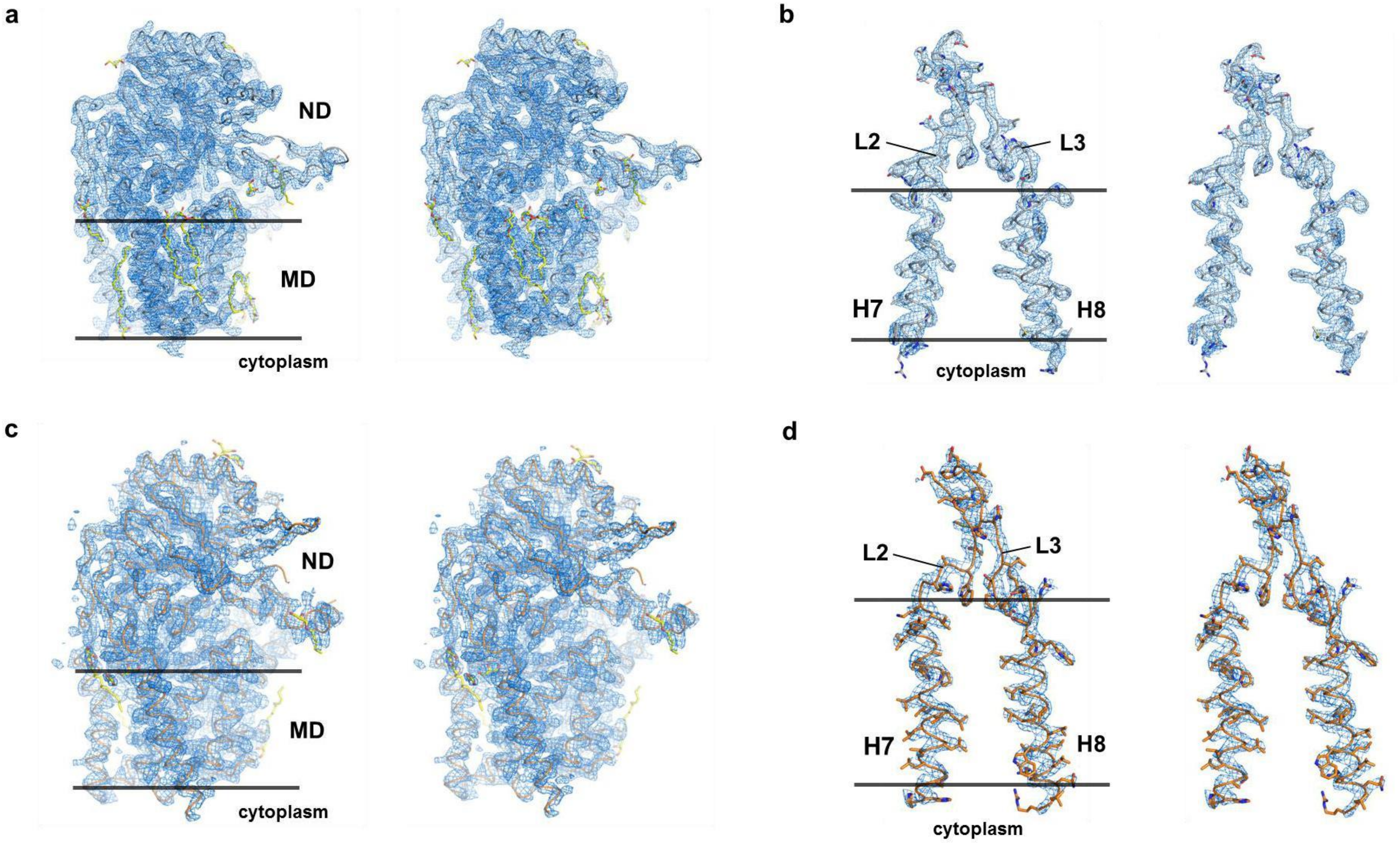

Supplementary Figure 2 | Stereo images of the structures of LntEco and LntPae WT. (**a**) 2Fo-Fc map of LntEco WT (gray) contoured at 1.5 σ (blue mesh). (**b**) 2Fo-Fc map of H7 and H8 and L2 and L3 in LntEco WT (gray) contoured at 1.5 σ (blue mesh). (**c**) 2Fo-Fc map of LntPae WT (orange) contoured at 1.5 σ (blue mesh). (**d**) 2Fo-Fc map of H7 and H8 and the L2 and L3 in LntPae WT (orange) contoured at 1.5 σ (blue mesh).

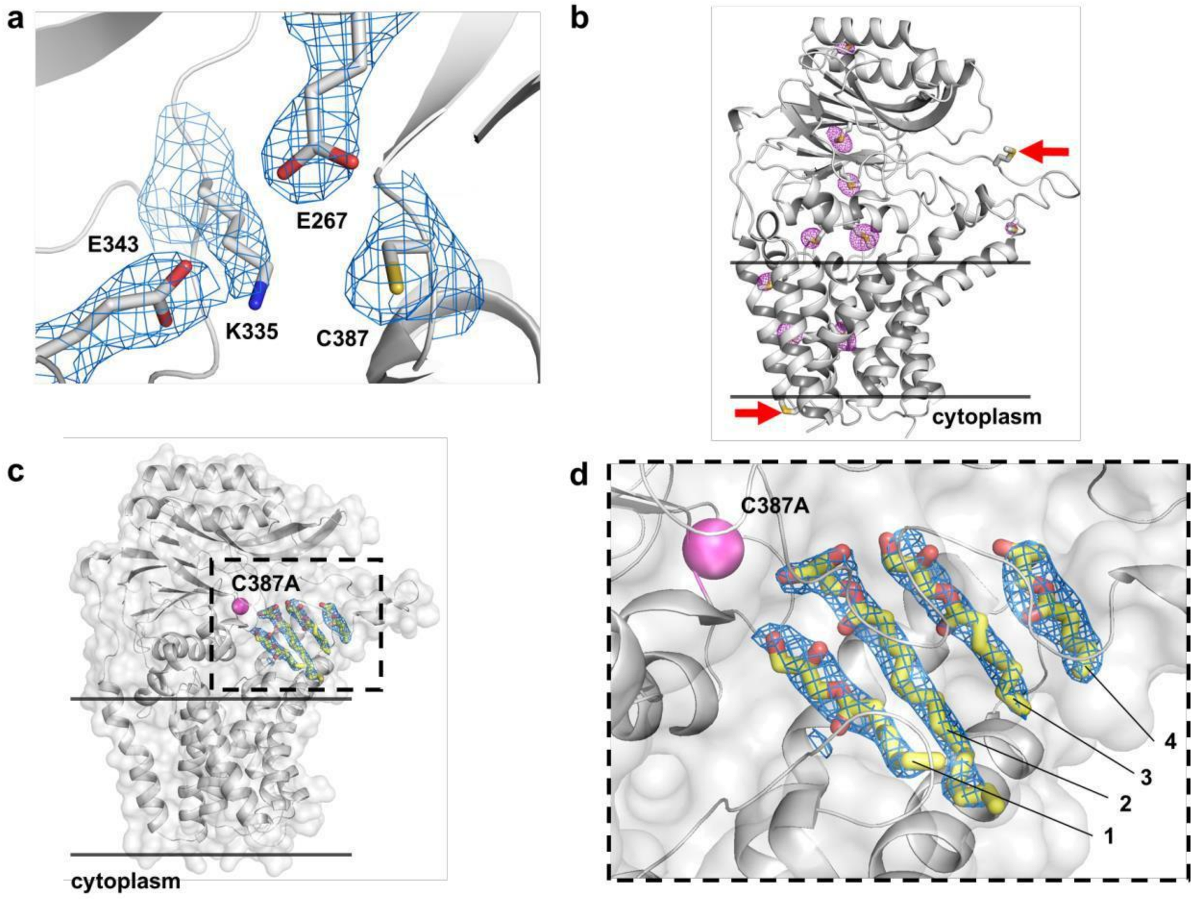

Supplementary Figure 3 | Electron density maps for LntEco. (**a**) 2Fo-Fc map of residues in the active site of LntEco contoured at 1.5 σ (blue mesh). Residue side chains are shown as thick sticks. (**b**) Selenium anomalous difference Fourier electron density peaks contoured at 4 σ (magenta net) are visible for 10 of the 12 methionine residues in LntEco. Methionine residues are represented as sticks. Red arrows identify methionines for which no anomalous difference density was observed. (**c**) Transparent surface representation of LntEco-C387A showing electron density modelled with monoolein lipid molecules in the portal leading to the active site. The 2Fo-Fc map is contoured at 1 σ (blue mesh) around the lipids shown as yellow sticks. C387A is represented as a sphere in magenta. (**d**) Expanded view of the boxed area in (c).

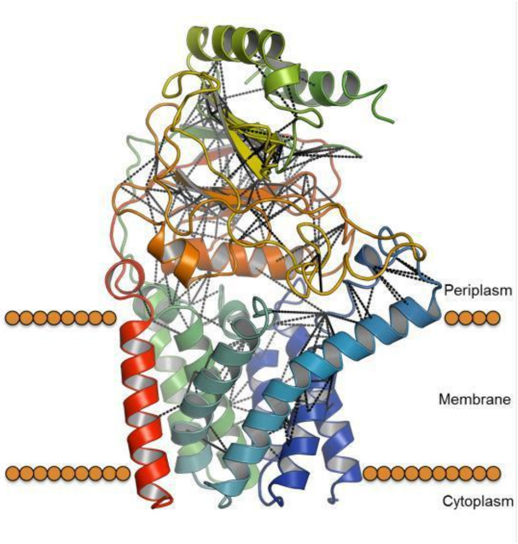

Supplementary Figure 4 | Co-evolutionary analysis. Co-evolutionary covariance analysis is consistent with the Lnt structures. For the most part, residue pairings predicted to exist in the native protein, based on a pattern of co-evolution, show up as proximal in the crystal structure. In the LntEco structure shown, predicted residue pairings are identified by dotted lines.

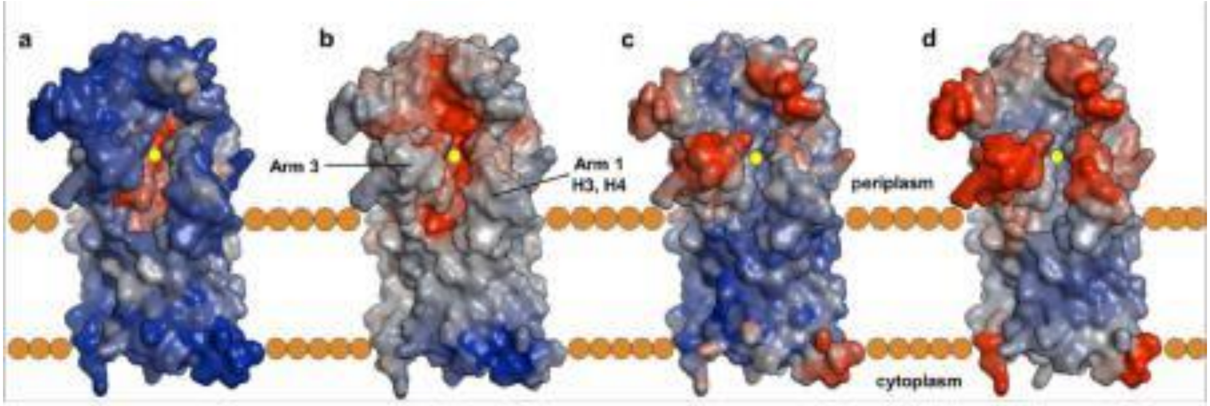

Supplementary Figure 5 | Assorted properties of LntEco in surface representation. (**a**) Conserved residues heat mapped onto structure demarks the putative active site pocket where the MD and ND come together. The view is from the membrane plane facing into the active site pocket. Catalytic triad residues locations are indicated. Conservation scale ranges from invariable (red) to non-conserved (blue). (**b**) Electrostatics range from -5 to 5 KT/e (red, negatively charged; grey, neutral; blue, positively charged). (**c**) X-ray B-factor values range from 150 $Å^2$ (red) to 30 $Å^2$ (blue). (**d**) Flexibility based on MDS on a blue to red scale from 0.5 to 3 Å RMSF. As a fiducial, a yellow dot is used to demark the approximate location of the catalytic Cys387. The crystallographic B-factor is an indicator of thermal motion. For residues in several of the arms surrounding the active site pocket the B-factor value is relatively high (**c**). This suggests that the arms have considerable flexibility, confirmed by MDS (**d**). Indeed, in certain instances electron density is missing in the loop regions consistent with an enhanced flexibility and disorder. Such flexibility may play a role in the mechanism of action of Lnt. The enzyme must function with a wide range of lipoprotein substrate types. It makes sense therefore that the arms at the front end of the molecule have the pliability and range of motion to accommodate the disparate physical and chemical features and dispositions with respect to the membrane of these substrates.

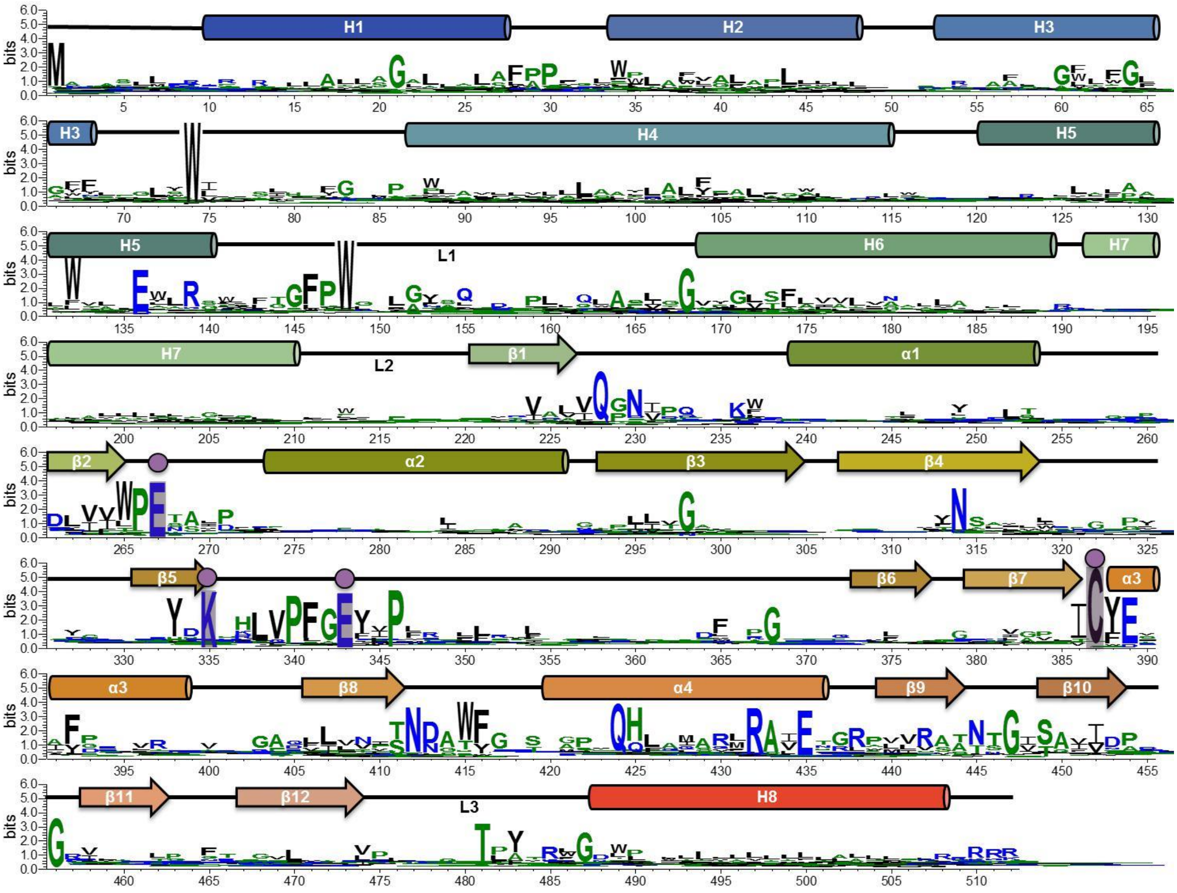

Supplementary Figure 6 | Sequence conservation and secondary structure of LntEco. A sequence Weblogo was constructed using the 1,116 Lnt sequences[1]. The four conserved residues in the active site, E267, K335, E343 and C387 are highlighted in purple. The secondary structure is shown above each sequence.

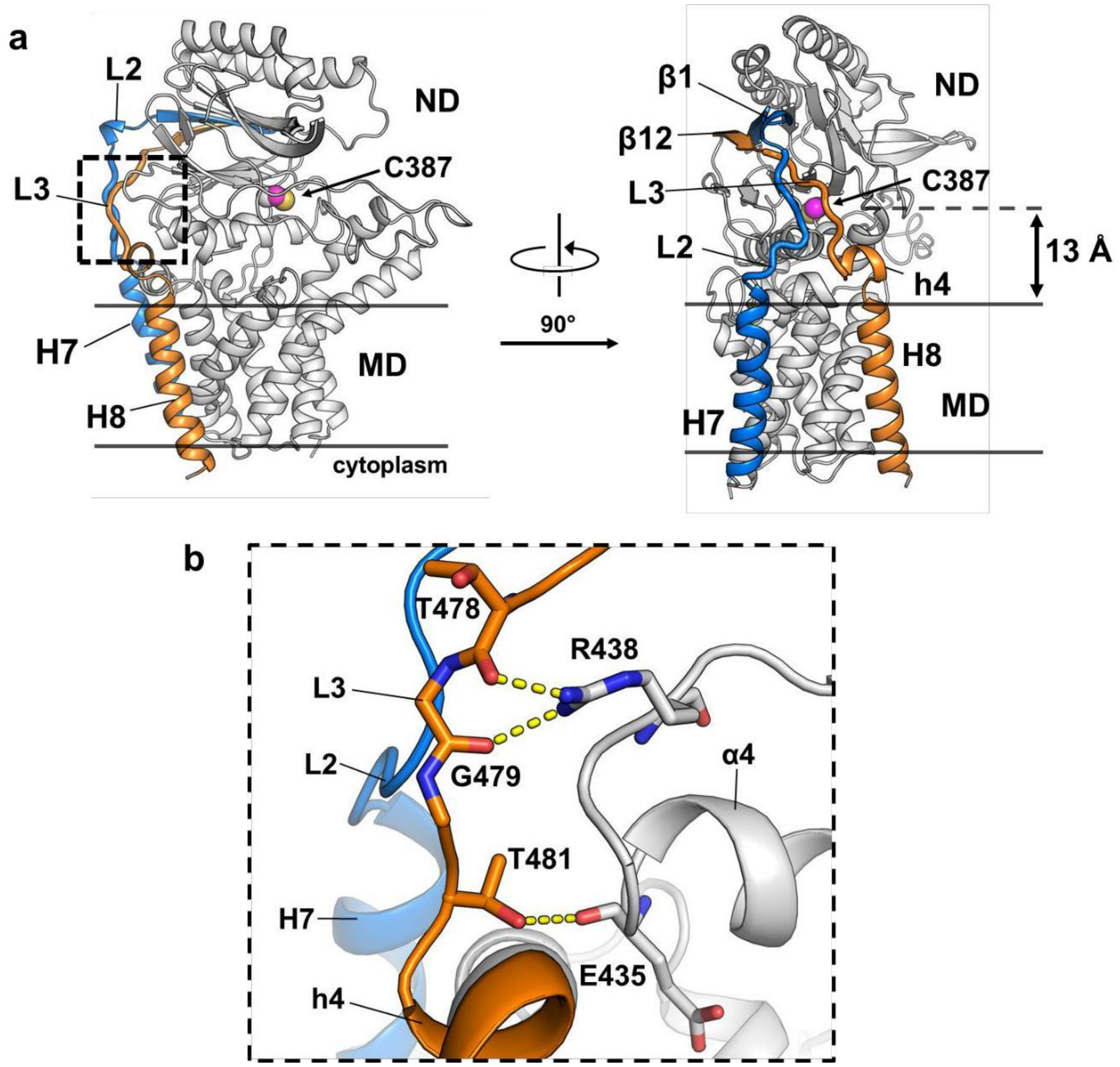

Supplementary Figure 7 | Linkages between the membrane and nitrilase-like domains. (**a**) Orthogonal views of LntEco from the membrane plane highlighting the connection between the MD and the ND by way of the brace-like linkers L2 (color blue) and L3 (color orange). (**b**) Coordination between residues in L3 and the ND help hold the two domains together. Conserved residues include Glu435 and Arg438. Gly479 and Thr478 is not conserved. Dashed lines correspond to distances ≤ 2.9 Å.

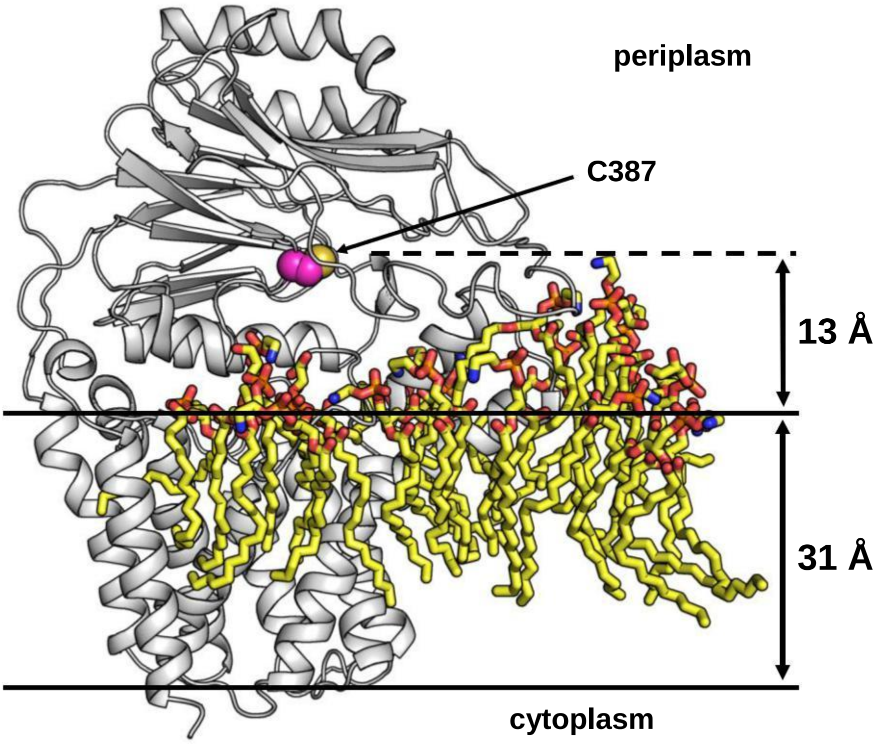

Supplementary Figure 8 | Lipids extend from the bulk membrane into substrate portal of LntEco.
An exemplar snapshot from a 100 ns MDS illustrating the protein stably situated in a membrane with phospholipid molecules rising out of the bulk membrane and infiltrating the portal leading to the active site pocket. Protein in cartoon representation viewed from the membrane plane. Lipid molecules in stick representation with yellow carbons. Cys387 shown as spheres.

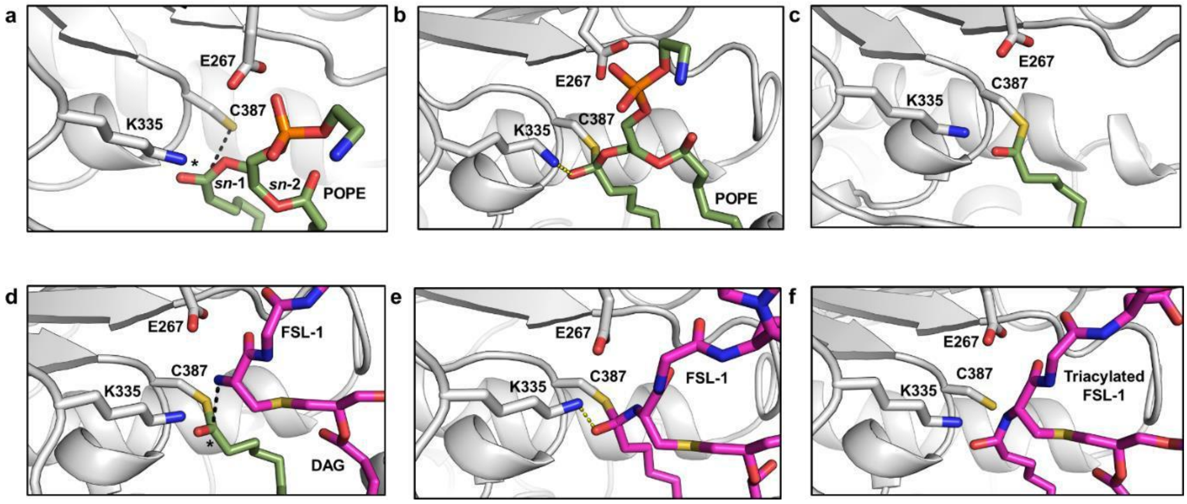

Supplementary Figure 9 | Recapitulating the N-acyltransferase reaction in the LntEco active site structure. Three-dimensional molecular models of the reaction cycle of LntEco, rationalised based on the coordinates of the monoolein lipid molecules bound in the LntEco-C387A structure. (**a**) Bound POPE substrate in close proximity to the catalytic residues, E267, K335 and C387. (**b**) Tetrahedral intermediate with POPE covalently linked to the C387 thiolate. (**c**) The *sn*-1 palmitoyl chain of POPE has been transferred to the S of C387 where it exists in thioether likage as the acyl-enzyme intermediate. (**d**) The diacylated lipoprotein substrate binds adjacent to the palmitoylated cysteine. (**e**) The lipoprotein tetrahedral intermediate includes covalent links to the lipoprotein N-terminus and to the palmitoylated C387. (**f**) The triacylated lipoprotein product of the reaction is released for the enzyme to reset. Dashed black lines in (a) and (d) indicate atoms in reacting partners that will end up being bonded together in the tetrahedral intermediate. The asterisk in (a) and (d) indicate carbon atoms that will become tetrahedral carbons in the tetrahedral intermediates. The dashed line in (b) and (e) indicate possible oxyanion stabilization by catalytic residue K335.

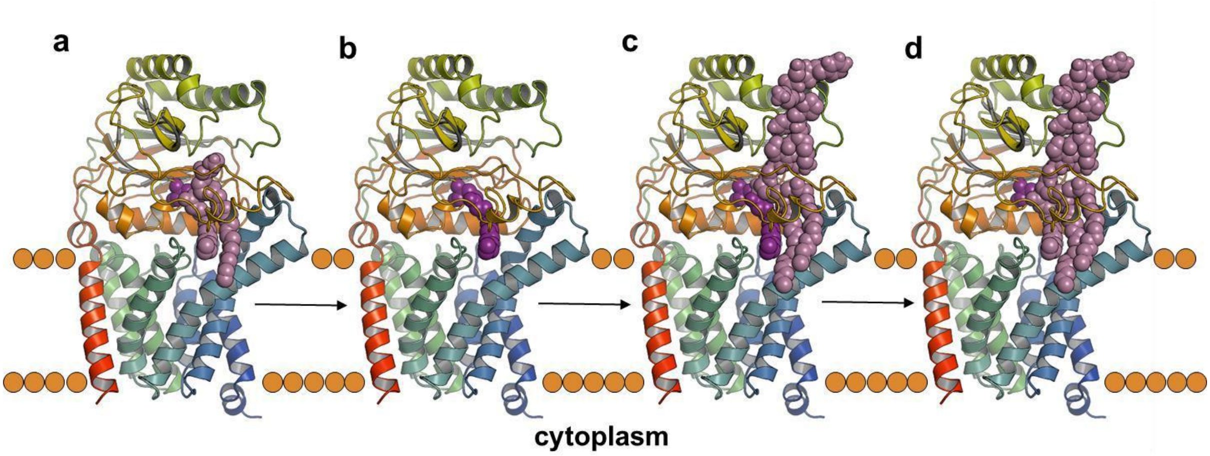

Supplementary Figure 10 | Overview of the molecular modelling of LntEco through the N-acyltransferase reaction. (**a**) The Michaelis complex of Lnt with POPE bound (purple spheres). (**b**) The palmitoylated-enzyme intermediate. A single acyl tail is transferred from POPE to the catalytic C387 residue. (**c**) Diacyl FSL-1 substrate (pink spheres) binds to the palmitoylated state of the Lnt enzyme as a second Michaelis complex. (**d**) FSL-1 is converted to its triacylated form through transfer of the palmitoyl from the enzyme. See also **Supplementary Movie 2**.

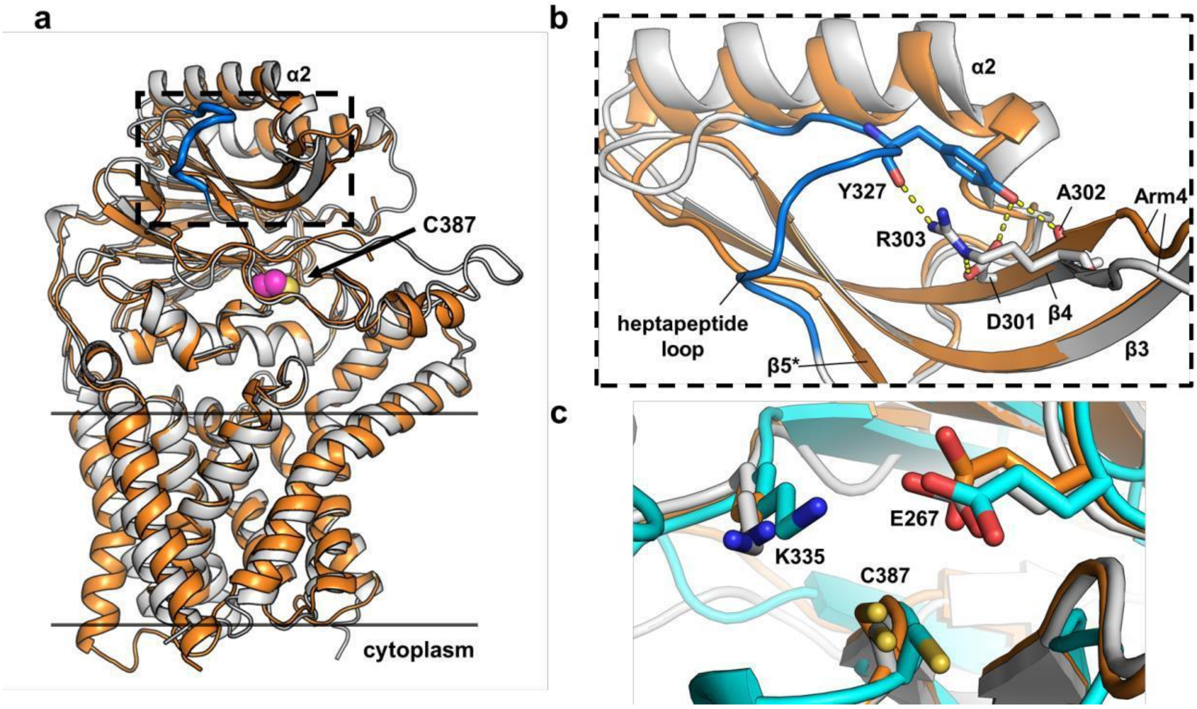

Supplementary Figure 11 | Structure homology of Lnt and catalytic triad residues. (**a**) Superposition of LntEco (grey) and LntPae (orange) models performed in PyMol. An heptapeptide loop in LntEco that is not present in LntPae is indicated in blue (boxed area). (**b**) An expanded view of the heptapeptide loop insert in (a) highlighting its interaction with the cleft created by 2, 3 and 4 of the ND. Coordinating distances of ≤ 3.1 Å are shown as dashed yellow lines. (**c**) Superposition of the catalytic triad residues in LntEco (grey), LntPae (orange) and mouse nitrilase-2 (cyan, PDB ID 2W1V). Catalytic triad residue side chains shown in stick representation.

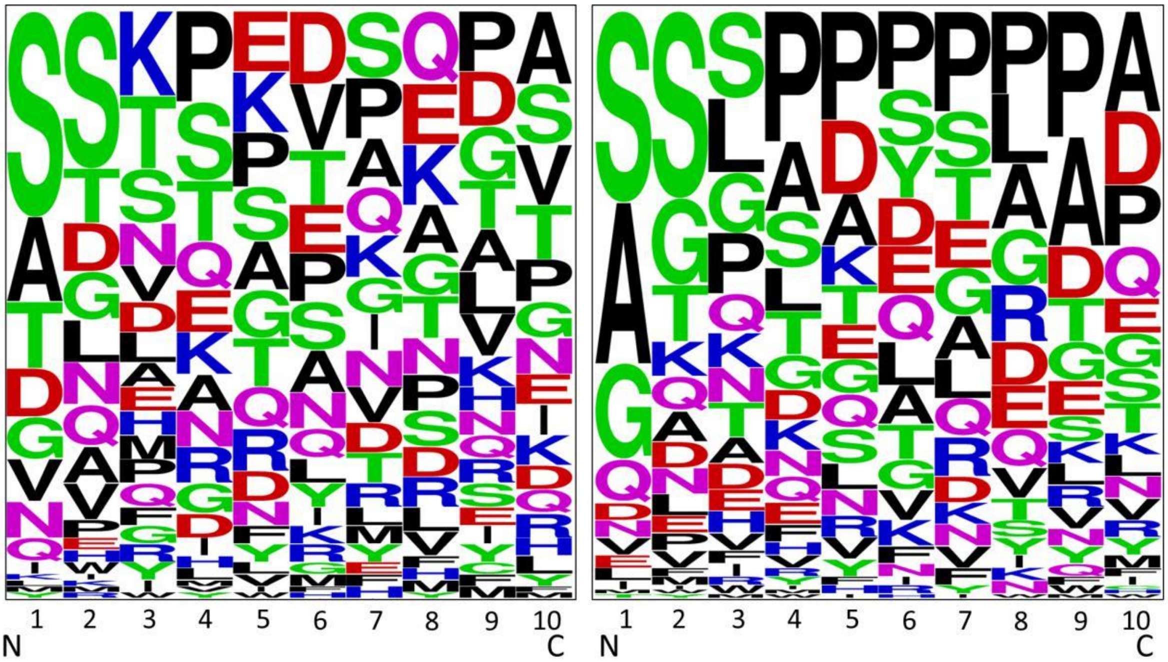

Supplementary Figure 12 | Residue frequency in the first eleven residues of lipoproteins from *E. coli* and *P. aeruginosa*. The N-terminal cysteine is invariant and is not shown. LOGO analysis[1] was carried out on 98 lipoproteins from *E. coli* (left) and 135 lipoproteins from *P. aeruginosa* (right). Lipoproteins were identified using an in-house *E. coli* and *P. aeruginosa* lipoprotein database assembled from sequences in UniProt[2]. All lipoprotein sequences were manually inspected to ensure they contained the appropriate lipoprotein signal sequence including the lipobox motif. Symbol size corresponds to the relative frequency of each amino acid at that position. The LOGO default color scheme was used: polar amino acids (G, S, T, Y), green; polar amino acids (N, Q), pink; basic amino acids (K, R, H), blue; acidic amino acids (D, E), red; hydrophobic amino acids (A, V, L, I, P, W, F, M), black.

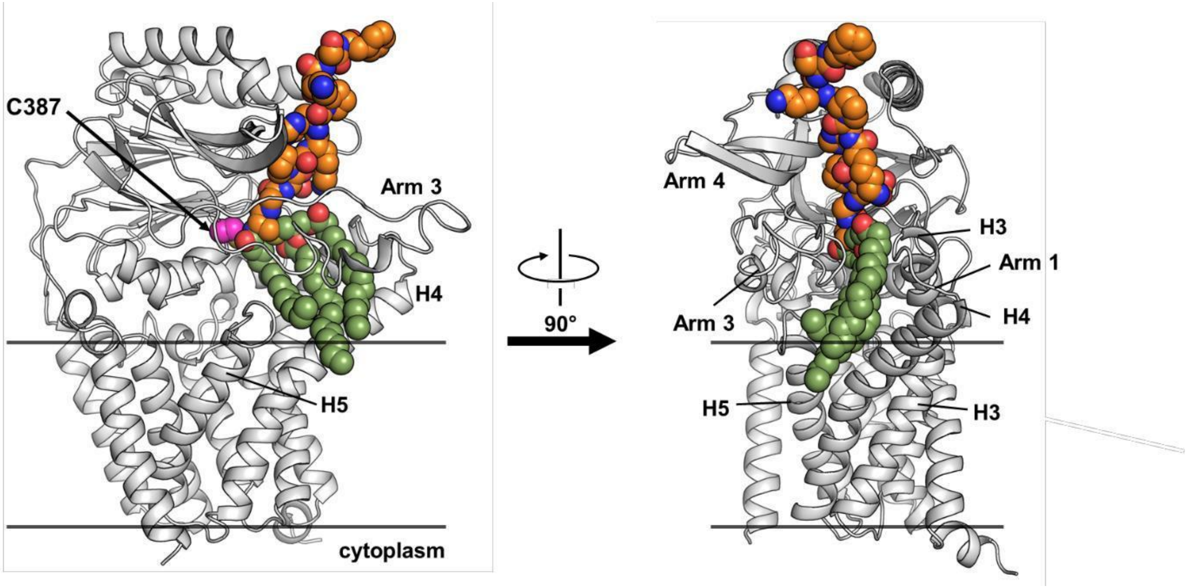

Supplementary Figure 13 | Unstructured peptide of FSL-1 is accommodated in the binding pocket of Lnt. Orthogonal views of LntEco (cartoon) from the membrane plane with the triacylated form of FSL-1 (spheres) modelled into the binding pocket. The decapeptide of FSL-1 was modelled as an extended, unstructured peptide and docked into the binding pocket with the acyl chains in the portal.

Supplementary Table 1 | pKa calculations performed on the Lnt X-ray structures using PROPKA. [3]
The residues included in the table are those highly conserved titratable residues, aligned for the two species for which we detail their X-ray structure. The residues in bold are those in the expected active site of the Lnt enzyme. The model pKa values for each residue species is shown in the last column.

LntEco		LntPae		Model
**Residue**	**pKa**	**Residue**	**pKa**	**pKa**
GLU136	5.38	GLU135	7.00	4.50
**GLU267**	**7.00**	**GLU269**	**6.82**	**4.50**
GLU343	3.97	GLU338	4.94	4.50
GLU389	5.83	GLU384	6.99	4.50
GLU435	6.21	GLU429	6.88	4.50
**LYS335**	**12.75**	**LYS330**	**13.56**	**10.50**
ARG432	14.80	ARG426	14.30	12.50
**HIS425**	**3.46**	**HIS419**	**3.66**	**6.50**
TYR333	16.27	TYR328	15.30	10.00
TYR388	13.55	TYR383	15.58	10.00
**CYS387**	**12.66**	**CYS382**	**13.04**	**9.00**

Supplementary Table 2 | Nucleotide sequences of the synthetic genes and primers used in this study.

**LntEco WT sequence**	ATGGGCAGCAGCCATCATCATCATCATCACAGCAGCGGCCTGGTGCCGC GCGGCAGCCATATGGCCTTCGCATCGCTGATTGAACGTCAACGCATTCG TCTGCTGCTGGCTCTGCTGTTTGGCGCATGTGGCACCCTGGCTTTTTCTCC GTACGATGTGTGGCCGGCGGCAATTATCAGCCTGATGGGTCTGCAAGCC CTGACCTTTAACCGTCGTCCGCTGCAAAGCGCGGCGATTGGCTTCTGCTG GGGCTTTGGTCTGTTCGGCAGTGGTATTAACTGGGTGTATGTTTCCATCG CAACGTTTGGCGGTATGCCGGGTCCGGTTAATATCTTCCTGGTGGTTCTG CTGGCGGCCTATCTGTCACTGTACACCGGCCTGTTTGCGGGTGTTCTGTC GCGTCTGTGGCCGAAAACCACGTGGCTGCGTGTCGCTATTGCAGCTCCG GCACTGTGGCAGGTTACGGAATTTCTGCGTGGCTGGGTCCTGACCGGTT TTCCGTGGCTGCAATTCGGCTACAGTCAAATCGATGGCCCGCTGAAAGG TCTGGCTCCGATTATGGGTGTGGAAGCGATCAACTTTCTGCTGATGATG GTGTCCGGTCTGCTGGCACTGGCACTGGTTAAGCGTAATTGGCGCCCGC TGGTCGTGGCAGTTGTCCTGTTTGCACTGCCGTTCCCGCTGCGCTATATT CAGTGGTTTACGCCGCAACCGGAAAAAACCATTCAAGTCAGCATGGTGC AAGGTGATATTCCGCAGTCTCTGAAATGGGACGAAGGCCAGCTGCTGAA CACGCTGAAGATTTATTACAATGCAACCGCTCCGCTGATGGGTAAAAGC AGCCTGATTATCTGGCCGGAAAGCGCTATTACCGATCTGGAAATCAACC AGCAACCGTTTCTGAAAGCACTGGATGGCGAACTGCGTGACAAGGGTA GTTCCCTGGTTACCGGCATTGTCGATGCCCGTCTGAACAAACAGAATCGC TATGACACCTACAATACGATTATCACCCTGGGCAAAGGTGCCCCGTATAG CTACGAAAGCGCGGATCGCTATAACAAGAATCATCTGGTCCCGTTTGGT GAATTTGTGCCGCTGGAATCTATTCTGCGTCCGCTGGCGCCGTTTTTCGA CCTGCCGATGTCATCGTTTAGTCGCGGTCCGTATATCCAACCGCCGCTGT CCGCCAATGGCATTGAACTGACCGCGGCGATTTGCTATGAAATTATCCTG GGCGAACAAGTGCGTGATAACTTCCGCCCGGATACGGACTATCTGCTGA CCATTTCAAATGACGCGTGGTTTGGCAAATCGATCGGTCCGTGGCAGCA CTTCCAAATGGCACGTATGCGCGCCCTGGAACTGGCACGTCCGCTGCTG CGCAGTACGAACAATGGTATTACCGCTGTTATCGGCCCGCAGGGTGAAA TTCAAGCGATGATCCCGCAGTTTACGCGTGAAGTGCTGACCACGAACGT TACCCCGACCACGGGTCTGACGCCGTATGCCCGTACCGGTAATTGGCCG CTGTGGGTCCTGACCGCACTGTTTGGCTTCGCAGCTGTGCTGATGAGCCT GCGTCAGCGTCGCAAATAA
**LntPae WT sequence**	ATGGGCAGCAGCCATCATCATCATCATCACAGCAGCGGCCTGGTGCCGC GCGGCAGCCATATGCGTTGGATTTCTCGTCCCGGCTGGCCCGGACACCT GCTGGCACTCGCCGCAGGCGCGCTCACACCCCTGGCCCTGGCGCCCTTC GACTACTGGCCGCTGGCGATCCTCTCGATCGCCCTGCTCTATCTCGGCTT GCGCGGCCTTCCCGGCAAGTCCGCCCTGTGGCGCGGCTGGTGGTACGGT TTCGGCGCGTTCGGCGCCGGCACCAGCTGGATCTACGTCAGTATCCATG ACTACGGCGCCGCCTCGGTGCCGCTGGCAAGCCTCCTCATGCTCGGCTTC ACCGCCGGCGTCGCCTTCTTCTTCGCCCTGCCGGCCTGGCTGTGGGCGC GCTGCCTGCGCCGCGACAACGCGCCGCTGGGCGACGCCCTGGCGTTCGC CGCCCTGTGGCTGGCACTGGAGCTGTTCCGCAGTTGGTTCCTTACCGGTT TCCCCTGGCTCTACGCCGGCTATAGCCAGTTGCAGGGACCGCTGGCCGG

	GCTGGTACCGGTGGGCGGCGTATGGCTGTCCTCCTTCGTCATCGCCCTCA<br>GCGCGGCCCTGCTGGTGAACCTGCCGCGCCTCTTCCCGCACGGTGCCAG<br>CCTGCTTCTGGGGCTGGTCCTGCTGCTCGGCCCGTGGGCGGCCGGCCTC<br>TATCTCAAGGGCCACGCCTGGACCCATTCGGCCGGCGAACCGCTGAGGG<br>TAGTGGCGATCCAGGGCAACATCGCCCAGGAGCTGAAATGGGACCCGA<br>ATCAGGTCCGCGCGCAACTCGACCTCTACCGCGACCTGAGCCTGCCGCA<br>GCAGGACGTCGACCTGATCGTCTGGCCGGAAACCGCGGTGCCGATTCTC<br>CAGGACATGGCCAGCGGCTACCTCGGCGCCATGGGCCAGGTCGCCGAC<br>GAGAAGAACGCCGCGCTGATCACCGGGGTCCCGGTGCGTGAACGTCTC<br>GCCGACGGCAAGAGTCGCTACTTCAACGGCATCACCGTGGTCGGCGAA<br>GGCGCGGGCACCTACCTCAAGCAGAAGCTGGTGCCGTTCGGCGAGTAC<br>GTGCCGCTGCAGGACCTGCTGCGCGGCCTGATCGCCTTCTTCGACCTGCC<br>GATGTCCGACTTCGCTCGCGGTCCGGCCGACCAGCCGCTGCTCAAGGCC<br>AAGGGCTACCAGATCGCCCCGTACATCTGCTACGAAGTGGTCTACCCGG<br>AGTTCGCCGCCGCGCTCGCGGCGCAGAGCCAGGTGTTGCTGACAGTGA<br>GCAACGACACCTGGTTCGGCACTTCCATAGGCCCTTTGCAGCACCTGCA<br>GATGGCACAGATGCGCGCCCTGGAAAGCGGTCGCTGGATGATCCGCGC<br>GACCAACAACGGCGTCACCGGCCTGATCGATCCCTATGGCCGGATCGTC<br>CGGCAGATCCCGCAGTTCCAGCAGGGCATCCTGCGCGGCGAGGTGATTC<br>CCATGCAGGGCCTGACGCCCTATCTGCAATACCGCGTCTGGCCGCTGGC<br>CGGGTTGGCCGGCGTGCTGCTGCTATGGGCTCTGCTCGGCCGCCAGTTG<br>CGTCCGCAGGAGCGACGCCTGTTCGGCTGA
**Primer sequence:**<br>**Lnt Eco C387A forward**	gaactgaccgcggcgattgcctatgaaattatcctggg
**Primer sequence:**<br>**Lnt Eco C387A reverse**	cccaggataatttcataggcaatcgccgcggtcagttc

Supplementary References

1 Crooks, G. E., Hon, G., Chandonia, J. M. & Brenner, S. E. WebLogo: a sequence logo generator. *Genome Res.* **14**, 1188-1190, doi:10.1101/gr.849004 (2004).

2 Consortium, T. U. UniProt: the universal protein knowledgebase. *Nucleic Acids Res.* **45** D158-D169 (2017).

3 Søndergaard, C. R., Olsson, M. H. M., Rostkowski, M. & Jensen, J. H. Improved Treatment of Ligands and Coupling Effects in Empirical Calculation and Rationalization of pKa Values. *J. Chem. Theory Comput.* **7**, 2284-2295, doi:10.1021/ct200133y (2011).
